# Auditory-Cortex Short-Term Plasticity Induced by Selective Attention

**DOI:** 10.1155/2014/216731

**Published:** 2014-01-12

**Authors:** Iiro P. Jääskeläinen, Jyrki Ahveninen

**Affiliations:** ^1^Brain and Mind Laboratory, Department of Biomedical Engineering and Computational Science, Aalto University School of Science, Espoo, 00076 AALTO, Finland; ^2^Department of Radiology, Harvard Medical School and Athinoula A. Martinos Center for Biomedical Imaging, Massachusetts General Hospital, Charlestown, MA 02129, USA

## Abstract

The ability to concentrate on relevant sounds in the acoustic environment is crucial for everyday function and communication. Converging lines of evidence suggests that transient functional changes in auditory-cortex neurons, “short-term plasticity”, might explain this fundamental function. Under conditions of strongly focused attention, enhanced processing of attended sounds can take place at very early latencies (~50 ms from sound onset) in primary auditory cortex and possibly even at earlier latencies in subcortical structures. More robust selective-attention short-term plasticity is manifested as modulation of responses peaking at ~100 ms from sound onset in functionally specialized nonprimary auditory-cortical areas by way of stimulus-specific reshaping of neuronal receptive fields that supports filtering of selectively attended sound features from task-irrelevant ones. Such effects have been shown to take effect in ~seconds following shifting of attentional focus. There are findings suggesting that the reshaping of neuronal receptive fields is even stronger at longer auditory-cortex response latencies (~300 ms from sound onset). These longer-latency short-term plasticity effects seem to build up more gradually, within tens of seconds after shifting the focus of attention. Importantly, some of the auditory-cortical short-term plasticity effects observed during selective attention predict enhancements in behaviorally measured sound discrimination performance.

## 1. Introduction

As so eloquently defined more than a century ago by philosopher William James, selective attention is “the taking possession by the mind, in clear and vivid form, of one out of what seem several simultaneously possible objects or trains of thought. Focalization, concentration, of consciousness are of its essence. It implies withdrawal from some things in order to deal effectively with others, and is a condition which has a real opposite in the confused, dazed, scatterbrained state” [1]. Subsequent behavioral research has elucidated the principles governing, for example, the role of memory in enabling selective attention in complex auditory scenes (see [2]). Elucidating the neural basis of the outright amazing ability to select task-relevant stimuli, including both external and internal ones such as memories and thoughts, and ignore task-irrelevant stimuli is one of the most fundamental research questions in cognitive neuroscience [3].

As will be reviewed in detail in the following, a number of recent findings have significantly shed light on the neural basis of selective attention. Specifically, it appears that selective attention is supported by short-term plasticity at the level of the auditory-cortex manifested as changes in neuronal receptive fields that filter attended sound features from amongst task-irrelevant ones. While some of these short-term plasticity effects seem to take place very quickly following a shift in the focus of attention, some seem to take longer to build up. Note that in line with our preceding work [4, 5], we here refer with the term short-term plasticity to any inputs, both excitatory and inhibitory, that transiently modulate the responsiveness of the target neurons to a subsequent stimulus. In order to place the findings on short-term plasticity in context, however, it is important to briefly appreciate how the auditory cortex is anatomically and functionally organized as that constitutes the framework within which selective-attention induced short-term plasticity operates.

## 2. Functional Neuroanatomy of the Auditory Cortices

While the detailed anatomical subdivisions of human auditory cortex have been more difficult to establish (e.g., using tonotopic mapping [6–20]) than in nonhuman primate models [21–28], it has been assumed that the primary auditory cortex resides in medial aspects of Heschl's gyrus (HG; *∼*Brodmann area 41 [29]) and is surrounded by nonprimary areas in anterolateral aspects of HG, superior temporal gyrus (STG), planum temporale (PT), and planum polare (PP) (*∼*Brodmann area 22). Overall, the nonprimary auditory cortices are more heterogeneous than the primary auditory-cortex, especially in terms of functional anatomy. Nonhuman primate models [24, 27, 28, 30, 31], human fMRI [32, 33], and human MEG [34–36] studies suggest that the primary core regions are responsive for higher stimulation rates and less complex sound features than the lateral nonprimary auditory cortices. Further, evidence for multiple tonotopically organized areas in the auditory cortices has been reported, which might reflect the presence of multiple functionally distinct areas, and stimulus feature selectivity has been observed in auditory cortex for sound periodicity [37, 38], location [39–42], and (constituents of) speech sounds [39, 43].

It has been further demonstrated that anterior and posterior nonprimary auditory-cortical areas show greater sensitivity to nonspatial and spatial sound attributes, respectively, in macaque monkeys [44] and cats [45]. Evidence for a broader division between parallel anterior “what” and posterior “where” pathways has been shown in several previous studies in humans [46–51], including a recent transcranial magnetic stimulation study [52], and this functional division seems to extend to higher-order regions in parietal and frontal lobes [53]. In addition to processing of auditory space, the dorsal pathway has been suggested to participate in auditory-motor integration and mediating of motor feedback to auditory areas (see [54, 55]).

Recent research has extended these findings by showing how “top-down” inputs during selective attention reshape the auditory-cortex neuronal receptive fields to help filter relevant stimulus features. In the present review, we focus on describing findings on such short-term plasticity phenomena; specifically how, when, and where top-down inputs modulate sound processing in the auditory system during attentive states, and how such short-term plasticity is associated with enhanced behavioral discrimination ability.

## 3. Primary Auditory System Short-Term Plasticity during Selective-Attention

One of the most central research questions in the neuroscience of auditory selective attention has been whether selective-attention modulates sound processing already in the primary auditory cortex, or whether the short-term plasticity caused by selective-attention is restricted to nonprimary auditory-cortical areas. Most of the earlier fMRI studies have simply probed whether significant hemodynamic response enhancements can be seen in nonprimary and primary auditory-cortical responses to sounds when they are selectively attended versus ignored. While some of these studies have provided evidence for the predominance of nonprimary auditory-cortex modulations [56–58], there are fMRI studies [59, 60] that, consistent with recent human depth-electrode cortical recordings [61], report also primary auditory-cortex modulation by selective attention. Thus, it seems that while nonprimary auditory cortex exhibits more robust modulation during selective listening, these effects do also involve the primary auditory cortex.

Observing selective-attention effects in primary auditory cortex in fMRI studies does not necessarily imply that selective-attention modulates the initial responses to auditory stimuli within this structure. Especially when using blocked-design paradigms, combined with the relatively low temporal accuracy of the blood-oxygenation level dependent responses that are measured with fMRI (*∼*seconds), the observed modulation of primary auditory-cortex responses could also be due to feedback inputs taking place at longer latencies. Thus, other methods, including electroencephalography (EEG) and magnetoencephalography (MEG), have been used to address the question of at which latencies selective-attention modulates processing of an incoming auditory stimulus.

In addition to being temporally accurate, MEG offers relatively good spatial localization accuracy, especially since the auditory-cortex is located mostly within the confines of the Sylvian fissure and thus the tangential component of the source currents (that is picked up by the MEG sensors [62]) is larger than the case of more radially oriented sources at the crowns of gyri (although see also [63]). Cortical folding along the length of the Sylvian fissure results in adjacent sources having different orientations, which makes the sources easier to separate with MEG inverse estimation. Therefore, MEG is rather optimally suited for studies of the human auditory-cortex and simultaneously collected EEG further helps disambiguate the underlying source configurations [64].

While the vast majority of MEG and EEG studies have documented selective-attention effects at latencies (and estimated cortical loci) beyond the initial responses that take place in primary auditory cortex (these findings are reviewed below), there are studies indicating that even the very early responses peaking ~50 ms from sound onset, and estimated to originate in the primary auditory cortex, are augmented by selective attention [65–67]. In these studies, responses to auditory stimuli when attended by experimental subjects in a highly focused manner have been compared with the responses to the same auditory stimuli when actively ignored by the subjects. Under such conditions, the amplitude of the early *∼*50 ms responses has been observed to be significantly enhanced, suggesting that processing of attended sounds is facilitated in primary auditory cortex. The precise mechanisms underlying the enhancement of these early-latency mass-action level responses during selective attention, however, remain an open question.

Augmentation of the initial primary auditory-cortex responses by selective attention raises the interesting question of whether the attended auditory stimuli are prioritized already at the level of subcortical auditory nuclei. Anatomically, this would be certainly possible via corticofugal connections that connect corresponding parts of the tonotopic maps, as documented in animal models [68, 69]. Overall, corticofugal connections do reach the subcortical auditory structures via fewer synapses than the ascending pathway reaches the auditory cortex, potentially allowing fast modulations upon changes in attentional focus, and the number of corticofugal connections is an order of magnitude larger than the number of ascending connections [70].

Overall, it has not been well established to date whether signal enhancements induced by selective attention extend to subcortical auditory structures in addition to auditory cortex in humans. Despite the negative results concerning brainstem auditory evoked potentials [71–73], evidence for attentional modulation of human peripheral auditory pathway has been found in EEG studies of brainstem frequency-following responses (FFR) [74, 75], including a recent study showing that the subcortical FFR to task-irrelevant sound features are suppressed when attention is being strongly directed to other sound features [76]. Selective-attention effects have been documented in recordings of otoacoustic emissions, that is, weak sound-signals emitted by the cochlea [77] though, again, there is also a very well conducted study where little selective-attention effects were seen at the level of cochlea [78].

It is possible that these discrepancies in findings are explained by the relatively small influence of attention on subcortical processing, combined with the fact that attentional effects depend on the rate of stimulation [79] and that there are fluctuations in attentional state during selective-attention paradigms [80]. In the visual modality, however, even larger selective-attention effects have been reported at the level of lateral geniculate nucleus of thalamus than primary visual cortex [81, 82], which suggest that subcortical modulations can play a crucial role in how selective-attention filters task-relevant information for further processing. Findings of plastic changes in subcortical auditory nuclei in animal models [69] do lend support for human findings of subcortical selective-attention effects. Further studies are nevertheless needed to elucidate the potential roles of the cortical and subcortical effects in selective attention.

## 4. Short-Term Plasticity in Nonprimary Auditory-Cortical Areas

While there have been relatively few studies describing early-latency selective-attention effects in primary auditory-cortical areas and subcortical structures, modulation of responses originating in nonprimary auditory-cortical structures in slightly longer latencies (from *∼*100 ms) has been widely documented, suggesting that selective-attention does induce the most robust short-term plasticity effects in nonprimary auditory-cortical areas. There are fMRI [56, 57, 83, 84], EEG [65, 85], MEG [86, 87], and multimodal spatiotemporal brain imaging [88] studies that have reported robust selective-attention modulation of nonprimary auditory-cortical areas.

The vast majority of these studies have documented enhancement of responses to sounds when they are attended versus ignored, making it difficult to make any inferences about the underlying neural mechanisms. There are, however, recent lines of research that have attempted to elucidate the underlying short-term plasticity mechanisms. One of these lines of research consists of studies documenting sound-feature specific response adaptation in specific cortical locations that can be interpreted as indicative of enhanced selectivity of the underlying neural receptive fields. Specifically, it is assumed in the adaptation studies that the degree of adaptation is governed by underlying neural selectivity. When two identical sounds are presented, the response to the latter sound is robustly suppressed. However, if the second sound of the pair differs from the first sound of the pair, release of adaptation is observed if the underlying neural population is selective to the sound feature that is different between the two sounds. For example, if the second sound differs in sound frequency from the first sound of the pair, release from adaptation is observed in cortical areas where the neurons are sharply tuned to respond to specific sound frequencies. Additionally, if selective attention to sound frequency enhances this release from adaptation as compared with the condition wherein the sounds are ignored, it can be inferred that selective-attention enhances tuning of receptive fields to the attended sound frequency.

**Figure 1:**
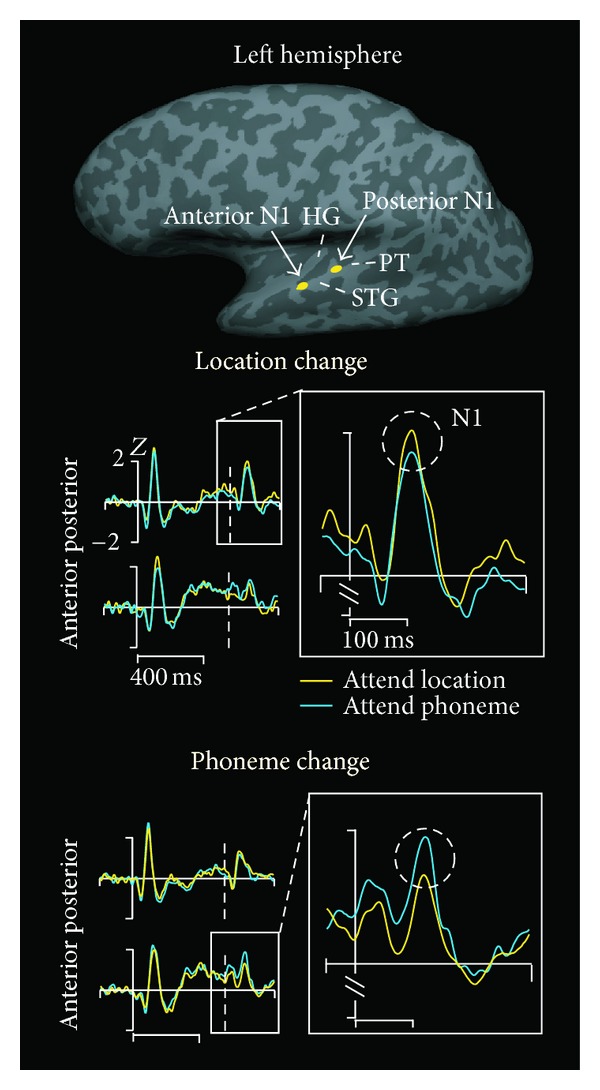
Task-specific attentional modulation of anterior and posterior auditory-cortex selectivity to phonetic category versus spatial location of sound source. Pairs of Finnish vowels /æ/ and /ø/ were presented from straight ahead or 45 degrees to the right. The stimuli were presented in pairs, adaptor followed by probe, which were spatially discordant, phonetically discordant, or identical. In attend location condition, subjects responded to sound pairs that matched the spatial pattern of the preceding sound pair (i.e., same sound source locations in the same order), irrespective of the phonetic content. In the attend phoneme condition, the targets were, in turn, sound pairs phonetically similar to the preceding sound pair (same phonemes in same order), irrespective of the spatial content. At the top is shown inflated left hemisphere with the locations of the anterior and posterior N1 sources (i.e., responses elicited *∼*100 ms from sound onset). As can be seen in the middle panel, the posterior N1 response amplitude to the probe following a spatially different adaptor stimulus was enhanced when subjects selectively attended spatial cues. Conversely, as seen in the bottom panel, anterior N1 activity to probes following phonetically different adaptor stimuli was enhanced by phonetic attention. This task- and cortical-location-specific reduction in a paired-stimulus adaptation paradigm suggested that neural selectivity to phonemes was increased in anterior auditory-cortex areas during selective attention to phonetic features, and that neural selectivity to spatial locations was increased in posterior nonprimary auditory-cortex during spatial selective-attention. These effects further occurred relatively rapidly, since the task changed once every 60 s (adapted with permission from [39]; HG: Heschl's gyrus; PT: planum temporale; STG: superior temporal gyrus).

The adaptation paradigm was utilized in a human neuroimaging study combining magnetic resonance imaging and MEG [39]. Adaptor-and-probe sound pairs were presented so that the adaptor and probe were either identical, or differed in phonetic category (Finnish vowel /æ/ versus /ø/), spatial location (0 versus 45 degrees to the right), or both. The degree of adaptation was then estimated across auditory-cortical locations, with reduced adaptation hypothesized to take place in cortical locations wherein the receptive fields of the underlying neural populations are selective to the respective auditory feature. As shown in Figure 1, it was observed that enhanced release from adaptation was observed in posterior nonprimary auditory-cortical areas when the probe and the adaptor differed in spatial location and, conversely, enhanced release from adaptation was observed in anterior nonprimary auditory-cortical areas when the probe and the adaptor sound differed in phonetic category. These results suggested that attention can enhance selectivity for sound identity and spatial location in the anterior and posterior nonprimary auditory-cortical “what” and “where” processing streams [39]. Analogous effects were found in a subsequent adaptation of fMRI study, which provided indices of attentional modulation of neuronal adaptation in certain auditory-cortex subregions sensitive to spatial versus sound identity features [56].

There are two alternative neural mechanisms that have been postulated to underlie enhancement by selective attention of sound-feature selectivity in specific auditory-cortical areas. The first of the hypothesized mechanisms is amplification of gain for processing attended and suppression of processing of unattended sounds without any modulation of neuronal receptive field properties, similarly to what has been reported in the visual modality [89]. The second hypothesis goes further in the extent of short-term plasticity that is assumed. According to the second hypothesized mechanism, receptive fields of auditory-cortical neurons are reshaped by attention to be more selective to features of the attended auditory stimuli, thus effectively filtering attended features from irrelevant auditory stimuli. This latter mechanism was also suggested to underlie effects shown in Figure 1 above. In the following, findings from human studies and animal models are reviewed that are relevant for these two hypotheses.

## 5. Gain Enhancement and Receptive-Field Reshaping as Potential Mechanisms

In order to decide between the alternative hypotheses of gain enhancement and receptive-field modulation, studies have been conducted where the shape of the neuronal receptive fields has been estimated using parametrically varying stimulation. Specifically, by presenting adaptor stimuli that parametrically vary from subsequently presented “probe” (or “test”) sounds along a single sound-feature dimension, it is possible to estimate the average shape of the receptive field of the underlying neural population [90]. The increased gain in such estimates is then expected to show up as multiplicative increase in response strength as a function of increasing distance in feature space between the adaptor and the test sounds in the selective-attention condition as compared with the ignore condition. Significant deviation from this expected effect could then be interpreted as indicating reshaping of the underlying neuronal receptive fields [91].

**Figure 2:**
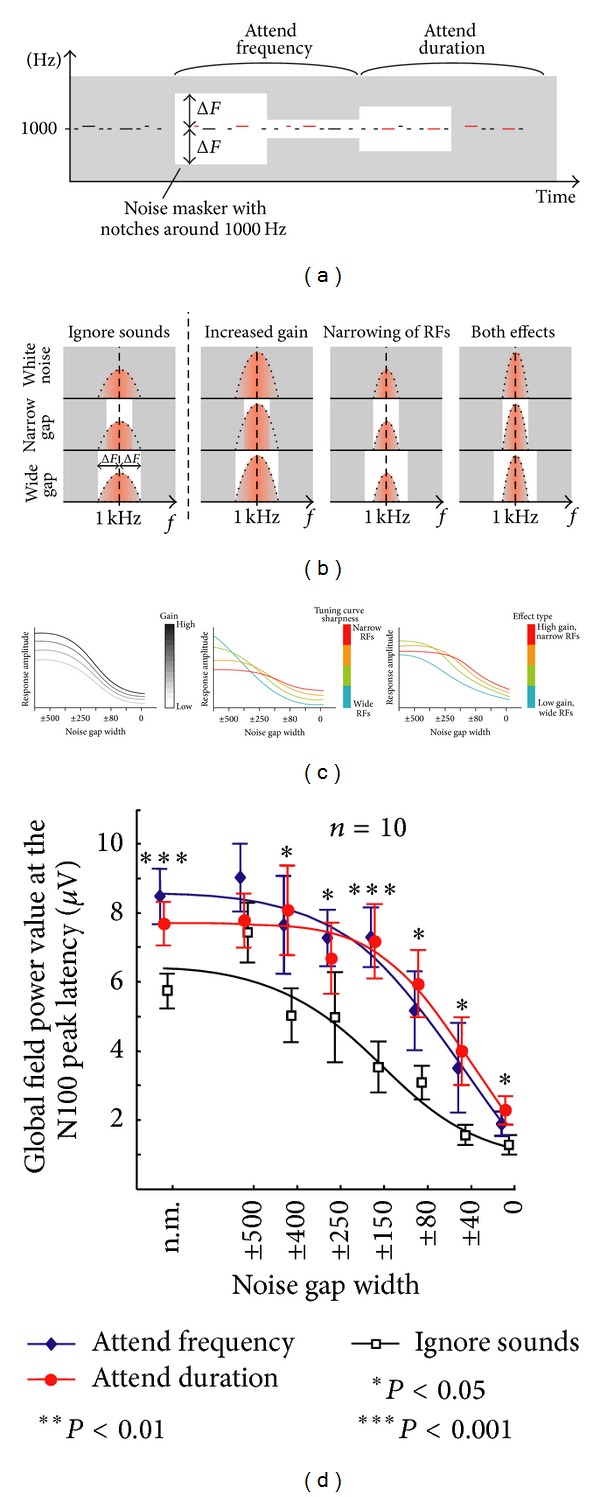
Selective attention increases both the gain and selectivity of auditory-cortex neural populations. (a) Target tones (red color) were either higher in frequency or longer in duration. Background gray represents the noise masker and the white area represents notch in the noise. (b) Bell-shaped curves represent the presumed single-neuron receptive fields (RFs) during baseline (Ignore) and the proposed attention-dependent changes in the RFs (increased gain versus narrowing of RFs versus both effects). It is assumed that noise suppresses responsiveness of the neuron to the 1 kHz tone as a function of its overlap with the receptive field of auditory-cortical neuron, with the red-colored area below the bell-shaped curve indicating how likely the neuron is to respond to the 1 kHz probe sound. In the white-noise condition, it is assumed that only neurons optimally tuned to the tone respond. (c) Simulated effects at the level of neuronal population responses as a function of notch width. Note that with the “gain only” mechanism, the amplitude-reduction function remains identical between the stimuli endpoints and is only scaled differentially, while the other mechanisms result in modulation of the basic shape of the amplitude reduction function. (d) Amplitudes at N1 response peak latency showed nonmultiplicative suppression with narrowing of the notch in the noise masker during selective-attention. Comparison with the three alternative models suggested that both increased gain and enhanced selectivity take place during auditory selective-attention. Adapted with permission from [91].

To test between these two alternative hypotheses, we presented in one of our studies 1 kHz sounds, embedded within notch-filtered noise masks with parametrically varying notch widths, to healthy volunteers during EEG recording [91]. By comparing the response adaptations as a function of notch width during states of selective attention versus ignoring, it was observed that adaptation of the global field power of time-averaged EEG responses at *∼*100 ms from sound onset was best explained by a model combining increased gain and enhanced tuning (see Figure 2). The spatial localization accuracy of EEG is, however, relatively low and thus it was not possible to determine decisively whether the observed short-term plasticity effects originated from the auditory-cortical areas, or whether, for example, putative frontal cortical contributions to the N100 response measured with EEG [92] contributed to the findings. MEG, offering better spatial localization accuracy than EEG, has been utilized in subsequent studies to show that there is either a combination of increased gain and receptive-field reshaping [93, 94] or relatively pure receptive-field reshaping effects [88, 95] that modulate, during selective-attention, the auditory-cortical response that is elicited *∼*100 ms from sound onset. Importantly, these short-term plasticity effects have been observed to correlate with behavioral discrimination accuracy [88, 91, 93].

Support for these human noninvasive EEG and MEG findings has been provided by research on animal models. Studies performed on awake ferrets, where sustained firing of single primary auditory-cortex neurons during presentation of so-called temporally orthogonal ripple combination sounds has been recorded to derive estimates of spectrotemporal receptive fields under baseline and attention conditions, have provided evidence of robust short-term plasticity of primary auditory-cortex neuronal receptive fields that further correlates with behavioral discrimination accuracy of the animals [96–100]. Furthermore, human MEG findings have recently suggested that there is even more robust tuning of auditory-cortical neuronal receptive fields at longer latencies of *∼*300 ms compared to the effects seen to take place at *∼*100 ms [93]. These longer-latency short-term plasticity effects were estimated to take place more medially (and slightly more anterior) compared with the posterior nonprimary auditory-cortical areas that were estimated to give rise to the *∼*100 ms response.

Interestingly, in the context of studies of selective-attention effects in visual cortical areas, it has been recently proposed that simple gain increase could take place when there are no competing stimuli within the receptive field of a neuron, and that reshaping of the receptive fields would take place when two or more stimuli occupy the neuronal receptive field [101]. In auditory studies, the procedure whereby adaptor sounds are utilized to probe the neuronal receptive fields naturally gives rise to circumstances where the adaptor (or notch-filtered noise masker) and probe/test sounds fall on the same neuronal receptive field, thus potentially explaining why short-term plasticity of the receptive field has been more readily seen in auditory studies. For analogous findings in visual cortex, see [102]. Interestingly, in recent intracranial recordings in humans, enhanced auditory-cortex responses to high-frequency sounds of an attended speaker were observed with concomitant suppression of responses for similar sounds in the to-be-ignored masker speaker [103].

## 6. Time Course of Selective-Attention Short-Term Plasticity Effects 

The time required for the short-term plasticity to take effect following shift in the focus of attention constitutes one of the most important questions when considering the behavioral relevance of the various types of short-term plasticity effects that have been associated with selective-attention. For example, if a given effect takes tens of seconds to build up, it can be assumed to play a rather different role in selective-attention than effects that are more or less instantaneous. While instantaneous effects might be associated with one's ability to rapidly shift attention between “attentional channels,” or from one perceptual object to another, effects with slower built-up might underlie fine-tuning or adaptation to a given sound environment. A behavioral example of this phenomenon in humans is a rapid (up to a few minutes) recalibration of auditory perception to a new reverberant environment [104]. Sound distance perception can also improve after a few sound repetitions in a reverberant space [105]. Interacting top-down influences such as expectations of the auditory environment [106] and bottom-up influences of sound repetition [107] seem to suppress conscious perception of echoes in comparison to the direct sound, thus increasing the target sound/background contrast. As yet another example, an enduring shift in the perceived location of sound sources called the ventriloquism aftereffect can result after an exposure of a spatial mismatch a few degrees lasting for 20–30 min between the locations of acoustic and visual stimuli [108–110]. A similar transient aftereffect may also be observed after spatially disparate acoustic and tactile stimuli [111]. Note, however, that there are findings suggesting that the ventriloquism effect could be fairly automatic, requiring little deliberate attention towards the visual stimulus that adjusts auditory spatial perception [112].

**Figure 3:**
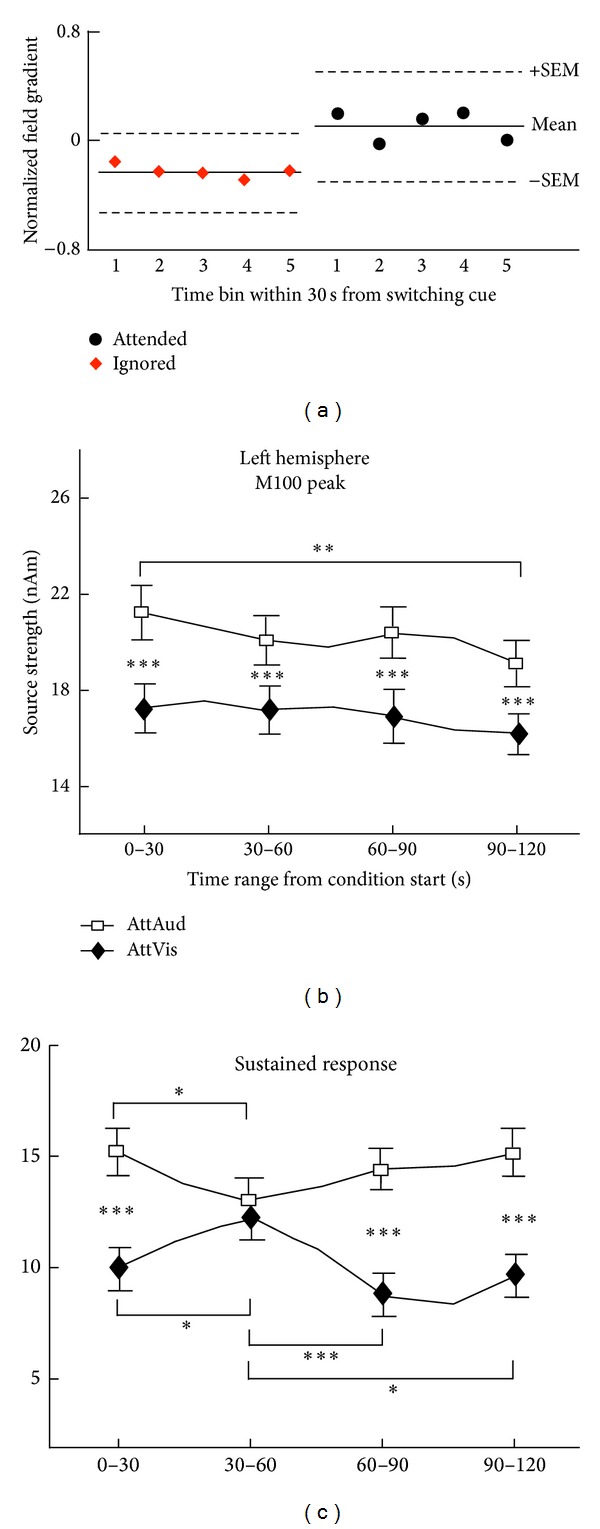
Time course of auditory-cortex short-term plasticity effects that take place during selective-attention. (a) Evolution of selective-attention effects within 30 s period that followed task-engagement, based on measurement of nonprimary auditory-cortical activity 50–150 from sound onset. The responses were allocated to five consecutive time bins and as can be seen the attention effects are observable already in the first responses (right, time bin 1) after attention switching, suggesting rapid (*∼*seconds) buildup of the short-term plasticity effects. Conversely, the similarity of response amplitudes to unattended tones across the bins (on the left) suggests that attention-induced short-term plasticity effects are washed out very rapidly following disengagement of attention. (b)-(c) Transient (*∼*100 ms from sound onset) and sustained response (*∼*300 ms) amplitudes as a function of time from the onset of attentional condition. Note again how the *∼*100 ms response attention effect does not show any dynamics, but the sustained response shows a significant interaction effect with attention and time range from the onset of the attention shift, suggesting that the short-term plasticity that modulates processing at *∼*300 ms from sound onset builds up much more gradually than the effects seen in activity that is elicited *∼*100 ms from sound onset. ((a) and ((b)-(c)) are adapted with permission from [88, 93], resp.).

There have been relatively few studies that have attempted to address the time course of auditory attention in humans. It is possible that the limited signal-to-noise ratios of the temporally accurate EEG and MEG methods available in human studies have limited the number of attempts since the attention shift over multiple trials would have to be repeated tens of times. The results of a recent combined MEG/EEG-fMRI study suggested that nonprimary auditory-cortex neuronal receptive-field changes associated with selective attention take place as rapidly as during the first seconds following shift in the focus of attention [88]. Interestingly, subsequent MEG study confirmed the quick time course of emergence of the *∼*100 ms response tuning by selective attention and further suggested that the longer-latency *∼*300 ms effects develop more slowly, over the time course of several tens of seconds [93]. For an illustration of these effects, see Figure 3. These findings tentatively suggest that the nonprimary auditory-cortical short-term plasticity effects that are seen *∼*100 ms from stimulus onset are associated more with facilitating rapid shifting of the focus of selective-attention. In contrast, the longer-latency effects could be associated with slower-onset tuning effects, such as those observed in behavioral spatial hearing experiments where gradual adjustment to echo properties of a room have been documented [104].

The question of how quickly the selective-attention effects wear off following withdrawal of attentional focus is related to the question of how quickly the effects develop and can be seen as lingering effects in paradigms where there are alternating shifts in the focus of attention. It has been shown in animal studies that at least some of the receptive-field reshaping effects observed at the level of single primary auditory-cortex neurons linger for extended periods of time after cessation of the task performance [99]. Tentatively, such effects could potentially underlie transition from short-term sensory-cortical plasticity supporting selective attention to longer-term plasticity effects that support perceptual learning, and indeed, receptive-field plasticity following conditioning has been described in animal models that greatly resemble receptive-field modulation under conditions of selective attention; for reviews on this, see [4, 113].

## 7. Concluding Comments and Suggestions for Further Research

It has been shown in both animal models and human neuroimaging studies that selective attention can modulate processing of attended sounds across multiple latencies and at multiple levels of the auditory system. It seems that processing in nonprimary auditory-cortical areas is modulated more robustly during selective attention than in auditory core areas or subcortical auditory structures. Specifically, there is accumulating evidence suggesting that top-down inputs during selective attention stimulus feature specifically reshape the receptive fields of neurons within functionally specialized nonprimary auditory-cortical areas, thus effectively filtering attended sound features from amongst task-irrelevant ones. While the receptive-field reshaping effects that modulate processing at *∼*100 ms from sound onset appear to take effect nearly instantaneously, short-term plasticity that modulates processing of sounds at longer latencies seem to build up over much longer time scales of tens of seconds. Given that the short-term plasticity effects predict enhancements in behaviorally measured sound discrimination performance, it can be assumed that auditory-cortex short-term plasticity (at least partially) underlies the ability of humans to filter the concurrently most relevant stimuli from amongst the countless number of task-irrelevant stimuli. Further research is, however, needed to fully elucidate the relative functional roles of the effects that have been documented to take place during selective attention at the different levels of the human auditory system.
